# Methylation Analysis of CpG Islands in Pineapple *SERK1* Promoter

**DOI:** 10.3390/genes11040425

**Published:** 2020-04-15

**Authors:** Aiping Luan, Chengjie Chen, Tao Xie, Junhu He, Yehua He

**Affiliations:** 1 Tropical Crops Genetic Resources Institute of Chinese Academy of Tropical Agricultural Science, Haikou 571101, China; 2 College of Horticulture, South China Agricultural University, Guangzhou 510642, China

**Keywords:** pineapple, *AcSERK1*, embryonic cell-specific promoter, CpG islands, methylation

## Abstract

Somatic embryogenesis (SE) is a more rapid and controllable method for plant propagation than traditional breeding methods. However, it often suffers from limited efficiency. *SERK1* promotes SE in several plants, including pineapple (*Ananas comosus* L.). We investigate the embryonic cell-specific transcriptional regulation of *AcSERK1* by methylation analysis of CpG islands in *AcSERK1* regulatory sequences. This revealed differences in the methylation status of CpG islands between embryonic callus and non-embryonic callus; the methylation inhibitor 5-azaC increased *AcSERK1* expression and also accelerated SE. These findings indicate that the expression of *AcSERK1* is regulated epigenetically. This study lays the foundation for further analysis of epigenetic regulatory mechanisms that may enhance the efficiency of SE in pineapple and other plants.

## 1. Introduction

Pineapple (*Ananas comosus* L.) belongs to the *Bromeliaceae* family. Pineapple is self-sterile, and sucker propagation has been the most popular propagation method since it was first introduced into Europe. The sucker propagation method has several advantages, including simplicity and low-cost. However, there are also many disadvantages, such as low reproductive coefficient, prolonged production periods, and non-uniform growth and development [1]. Therefore, alternative breeding methods based on tissue culture have become a preferred choice for pineapple planting.

Somatic embryogenesis (SE) is an ideal rapid propagation method for many different types of plants and trees in vitro that also fits the requirements of industrialization [2]. However, it can suffer from limited efficiency. Therefore, a better understanding of the molecular mechanisms underlying SE may lead to the development of novel methods to improve efficiency. The physiological, biochemical, and morphological changes that underlie SE are regulated by many genes. SE can be divided into two stages. Stage one is the embryogenic induction stage, that is, an acquisition of the ability to undergo somatic embryogenesis. Stage two is the developmental stage of somatic embryos, including the process by which embryonic cells form mature embryos, which occurs via developmental processes similar to zygotic embryos. Most SE-related genes play a role in the later stages of somatic embryogenesis, such as *LEC1* [3], *LEC2* [4], *AGL15* [5], *BBM* [6], and *WU* [7], which all increase SE or maintain the ability of the cells to undergo SE. Only somatic embryogenesis receptor kinases (*SERKs*) have been found to play an important role in the earlier transition from somatic cells to embryonic cells [7,8,9]. This small gene family of receptor-like kinases plays diverse roles in plants [10,11]. Among the *SERK* family, *SERK1* is regarded as a SE marker gene of pineapple and other plants [12,13]. *SERK1* expression is associated with the induction of SE, and also promotes the transformation of somatic cells to embryonic cells. For instance, the expression of *SERK1* in *Arabidopsis thaliana* has been shown to increase its SE capacity by 3 to 4 times [14]. A similar phenomenon has also been reported in pineapple. The expression of *SERK1* in pineapple has been shown to increase its SE capacity by about two times [12].

SE-mediated plant regeneration during in vitro culture is a complex process involving the actions of hormones, transcription factors, and epigenetic regulators [15,16]. One major epigenetic modification is DNA methylation, which plays a key role in SE and plant regeneration [17]. However, the relationship between the methylation levels of CpG islands in the promoter of key genes, gene expression levels, and the process of SE is still unclear. In this study, we report differences in the methylation status of CpG islands in the *AcSERK1* regulatory sequence in embryonic versus non-embryonic callus from pineapple. We found that lower levels of DNA methylation at this locus were associated with higher expression, which may promote embryogenic competence during the in vitro culture of pineapple. The information provided here will form the foundation for future research on genetic and epigenetic control of plant SE during in vitro culture.

## 2. Materials and Methods

### 2.1. Plant Materials

The “Shenwan” pineapple species was used in this study. It was collected in 2013 from South China Agricultural University, Guangzhou, China. Suckers were obtained and cultured on Murashige and Skoog medium supplemented with 2 mg/L NAA and 3 mg/L BA to obtain callus [18].

For callus treatment, the proliferated callus was pre-cultured in liquid MS medium containing the methylation inhibitor 5-azaC [19] at 0.5, 5, 50, and 100 μmol·L^−1^ and for 3, 5, 7, and 9 days. 5-azaC was dissolved directly in the medium, therefore callus cultured in liquid MS medium without added 5-azaC served as a negative control (Control). Thereafter, the pre-treated-callus and negative control were transferred to embryonic induction medium (MS + 5 mg/L 2,4-D + 0.5 mg/L BA) at the same time to induce the production of embryonic callus [18]. The induction processes were both sampled every 5 days, quickly frozen in liquid nitrogen and stored in a −80 °C ultra-low temperature freezer to extract RNA for qPCR analysis to measure *AcSERK1* expression during somatic embryo induction.

### 2.2. Gene Expression Analysis

Total RNA was extracted using TRIzol reagent (Invitrogen, Shanghai, China) from each callus sample and PrimeScript^TM^ RT reagent kit with gDNA Eraser (TaKaRa, Dalian, China) was used to synthesize the corresponding cDNAs. These cDNAs underwent quantitative real-time PCR (qPCR) using Thunderbird SYBR qPCR Mix (Toyobo, Shanghai, China) in the iQ5 Real-Time PCR system (BioRad, Hercules, CA, USA). The primers used are listed in Table 1. Each reaction was performed in biological triplicates. The cycling conditions were as follows: 95 °C for 10 s, 94 °C for 5 s, 53 °C for 20 s, 40 cycles. The reaction systems were as follows: cDNA template 1 μL, mix primers (forward and reverse, 2.5 μmol·L^−1^) 2 μL, SYBR Mix 10 μL, ddH_2_O 7 μL. The pineapple *β-actin* gene was used as a reference gene, and expression levels were normalized to this gene [12]. Relative gene expression values were calculated using the 2^−∆∆CT^ method [12]. The expression differences between different treatments were compared using a one-way ANOVA in SPSS 19.0.

### 2.3. Methylation Analysis of the AcSERK1 Promoter Region

We used EMBOSS GUI v1.12: cpgplot (http://www.ebi.ac.uk/Tools/seqstats/emboss_cpgplot/) to predict the CpG islands in the regulatory sequences of *AcSERK1*, *2*, and *3*. Methprimer was used to design BSP (Bisulfite sequencing PCR) primers (Table 2) to amplify the CpG islands in the *AcSERK1* regulatory sequences. These primers are not bisulfite-specific primers but could also amplify non-bisulfite treated genomic DNA. The reverse primer amplifying CpG-2 island is strongly biased for hybridization to a complementary methylated bisulfite-treated DNA.

Bisulfite treatment was performed as described in the EZ DNA Methylation-Gold KiT^TM^ kit instructions. The main steps were as follows: 400 ng of genomic DNA was denatured at 98 °C for 10 min under CT Conversion Reagent transformation reagent, and vulcanized at 64 °C for 2.5 h, followed by desalting desulfurization, washing, and elution with 15 μL of eluate to recover the converted DNA. The reaction system of BSP amplification was as follows: bisulfite-treated template DNA 170 ng, forward primer (10 nmol·L^−1^) 1 μL, reverse primer (10 nmol·L^−1^) 1 μL, 2 × PCR Buffer 5 μL, dNTPs (1.25 mmol·L^−1^) 5 μL, ddH_2_O 5 μL. The cycling conditions of BSP amplification were as follows:
97 °C Pre-denaturation7 minHot-start Taq enzyme (5U·μL^−1^)0.55 μL97 °C Pre-denaturation7 min95 °C 1 min, 55 °C 1 min, 72 °C 1 min10 cycles95 °C 1 min, 53 °C 1 min, 72 °C 1 min10 cycles95 °C 1 min, 50 °C 1 min, 72 °C 1 min10 cycles95 °C 1 min, 48 °C 1 min, 72 °C 1 min10 cycles72 °C Extension10 min

The BSP amplification product was ligated into the TA cloning vector and transfected into competent *E. coli* DH5α, and positive clones were identified by blue/white screening. Using the white colony as a template, PCR amplification was performed using methylation-specific primers. Positive clones were identified by 1% agarose gel electrophoresis. These were inoculated into LB/Amp + liquid medium and cultured overnight at 37 °C on a shaker at 220 rpm. The plasmids were extracted using a biochemical plasmid kit and sent to Guangzhou Meiji Biotechnology Co., Ltd. for Sanger sequencing.

### 2.4. Methylation Inhibitor Pretreatment before Somatic Embryogenesis Induction

We selected the concentration and the treatment time of 5-azaC that resulted in the highest *AcSERK1* expression in the qPCR analysis as the optimal treatment conditions for 5-azaC pretreatment before embryonic induction. After somatic embryos were induced for 30 days, the somatic embryos arising from 5-azaC pre-treated callus and untreated callus (negative control) were observed microscopically, and the number of somatic embryos and adventitious buds were counted.

## 3. Results

### 3.1. Methylation Analysis of CpG Islands in the AcSERK1 Promoter Region

**Figure 1:**
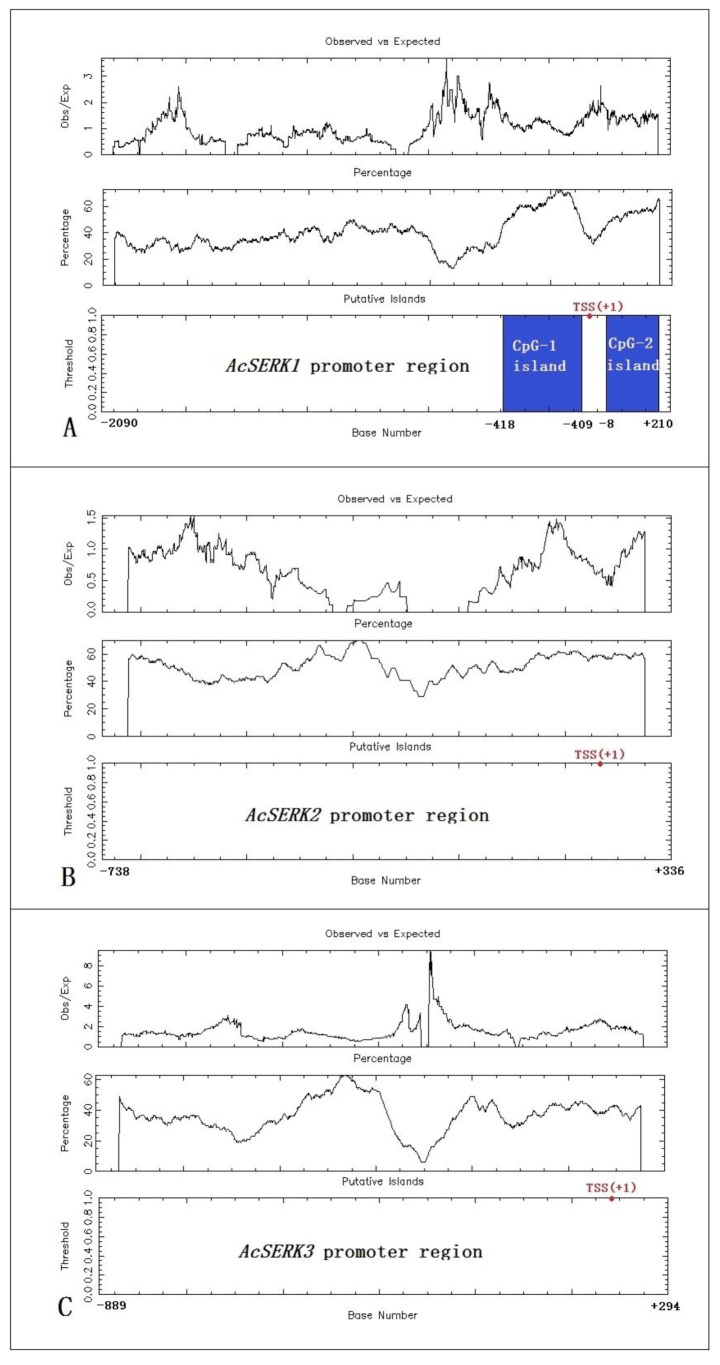
Bioinformatics prediction of CpG islands in the promoter regions of three *AcSERKs*. (**A**) *AcSERK1* promoter region contains two CpG islands, which are indicated in blue. (**B**,**C**) The bioinformatics prediction results of CpG islands in the *AcSERK2* and *AcSERK3* promoters, respectively. Neither is predicted to contain CpG islands. The Cpgplot program defines a CpG island as a region corresponding to the following parameters: Observed/Expected ratio >0.60, Percentage C + Percentage G >50.00, and Length >200. The transcription start site is represented by TSS (red dot), and this site is defined as +1.

CpG island predictions were performed on the regulatory sequences of the three *AcSERK* genes using EMBOSS GUI v1.12: cpgplot. *AcSERK1* is located on “Shenwan” pineapple chromosome 2. We identified two CpG islands in the promoter region. The CpG-1 island is located upstream of the transcription start site TSS (+1) and is 310 bp (−418 to −109). The CpG-2 island is located downstream of the TSS, but still within the designated promoter region, and is 219 bp (−8 to +210). *AcSERK2* is located on chromosome 1, and *AcSERK3* is located on chromosome 15. No CpG islands were predicted in their 5′ upstream regulatory sequences by EMBOSS GUI v1.12: cpgplot (Figure 1).

**Figure 2:**
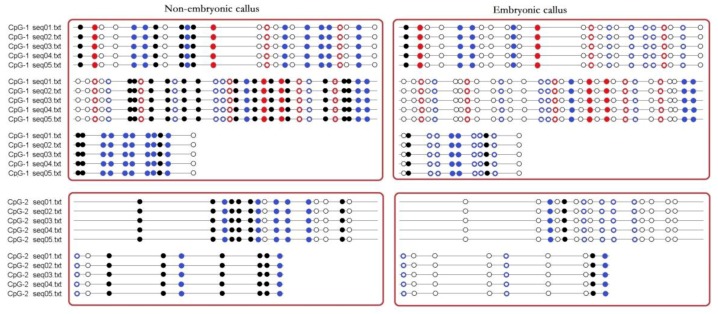
Schematic diagram of CpG promoter methylation sites in *AcSERK1*. Black dots represent methylated CG sites, and white dots represent unmethylated CG sites. Red dots represent CHG. Blue dots represent CHH. H = A, C, or T. Solid dots represent methylated sites. Hollow dots represent unmethylated sites. The results represent 5 biological replicates (labeled as seq01.txt to seq05.txt).

Although BSP primers could not exclude the amplification of non-bisulfite treated genomic DNA, the BSP sequencing results could still show the methylation status of the CpG island of the *AcSERK1* promoter. Methylation of different sites in the CpG islands of the 5′ upstream regulatory region of *AcSERK1* was observed in the embryonic and non-embryonic callus (Figure 2). Thirty-two CG, 11 CHG, and 23 CHH sites in the CpG-1 island were present. Of these, only 3 CG, 4 CHG, and 8 CHH sites were methylated in embryonic callus. In contrast, in non-embryonic callus, 20 CG, 4 CHG, and 18 CHH were methylated (Figure 2).

In the CpG-2 island, which was downstream of the TSS in the *AcSERK1* promoter, there were a total of 16 CG and 8 CHH sites. Of these, only 2 CG and 2 CHH sites were methylated in embryonic callus. In contrast, in non-embryonic callus, 11 CG and 7 CHH were methylated (Figure 2). Thus, the methylation status of the two CpG islands in the *AcSERK1* promoter during in vitro culture is differentially regulated between embryonic and non-embryonic callus.

### 3.2. Effect of Methylation on the Expression of AcSERK1

**Figure 3:**
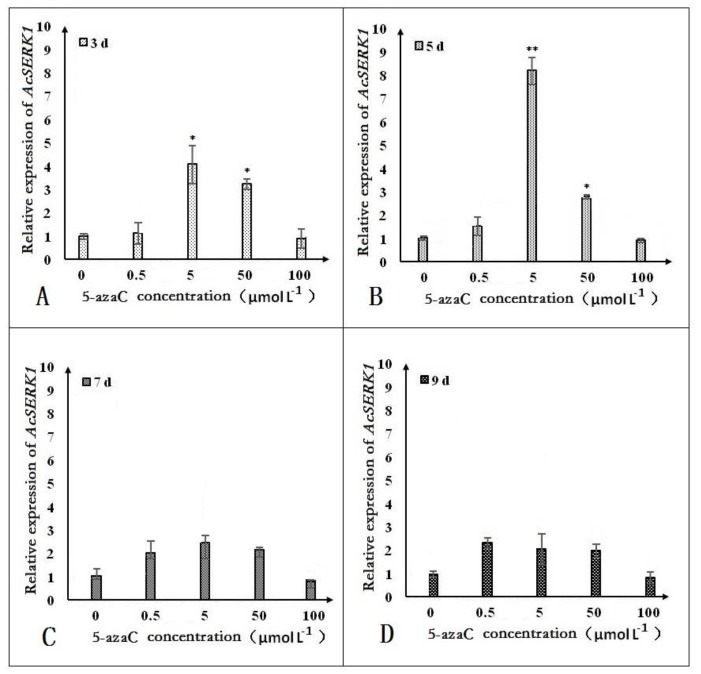
Effect of different concentrations of 5-azaC treatment for different times (3, 5, 7, or 9 days) on the expression levels of *AcSERK1*. (**A**–**D**) The effects of different concentrations on the expression of *AcSERK1* during 5-azaC pretreatment at 3, 5, 7, and 9 d, respectively. The data were the average of 3 biological replicates, and the error bars denote the standard error of the mean. The expression differences with different treatments were compared using a one-way ANOVA by SPSS 19.0. * *p* < 0.01 and ** *p* < 0.05.

To determine whether methylation of *AcSERK1* plays a role in its expression, we analyzed the expression pattern of *AcSERK1* in non-embryonic callus by qPCR with and without treatment with different concentrations of the methylation inhibitor 5-azaC for different durations. We found that the 0.5 μmol·L^−1^ pre-treatment for three or five days could not induce *AcSERK1* expression (Figure 3). The most dramatic effect of 5-azaC on *AcSERK1* expression was observed with 5 μmol·L^−1^ 5-azaC pretreatment for 5 days (Figure 3B). Briefly, 5 and 50 μmol·L^−1^ pre-treatments for 3 and 5 days were the most effective, with no statistical differences for longer pre-treatments (Figure 3A,B).

**Figure 4:**
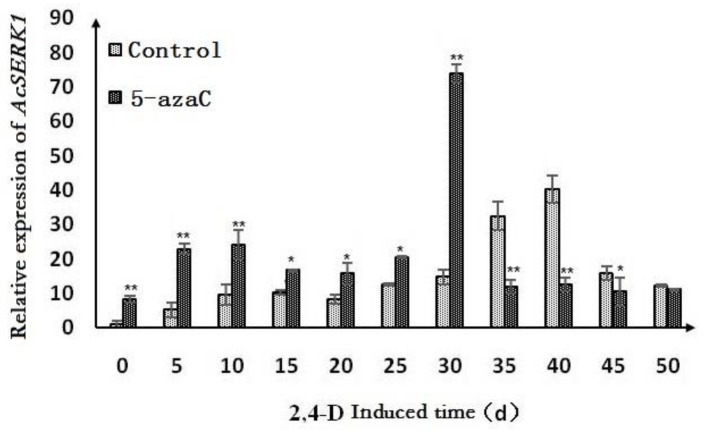
Effect of 5-azaC on the expression of *AcSERK1* during the process of SE. The data were the average of 3 biological replicates and the error bars denote standard error of the mean. Comparisons between pretreated (5-azaC) or untreated (“Control”—without 5-azaC) at the same induction times. The expression differences with different treatments were compared using a one-way ANOVA by SPSS 19.0. * *p* < 0.01 and ** *p* < 0.05.

To analyze the effect of methylation inhibition on *AcSERK1* expression in embryonic callus, we transferred callus to liquid MS medium containing 5 μmol·L^−1^ 5-azaC and suspended it for 5 days before embryonic induction. We chose these conditions as they induced the highest increase in expression in the non-embryonic callus. During SE induced by 2,4-D, there were differences in the expression patterns of pineapple callus pre-treated with 5-azaC or without pretreatment (Figure 4). In the absence of 5-azaC pretreatment, the expression of *AcSERK1* in callus induced by 2,4-D was consistent with published results [12]: increasing slowly from day 10 to peak at day 40, before decreasing. However, after 5-azaC pretreatment, the expression of *AcSERK1* in the callus induced by 2,4-D increased more rapidly (already at day 5 after induction, with an earlier peak at day 30) before decreasing (Figure 4). Thus, methylation inhibition led to a more rapid onset of *AcSERK1* expression during somatic embryo induction.

### 3.3. Effect of Methylation Inhibition on SE in Pineapple

**Figure 5:**
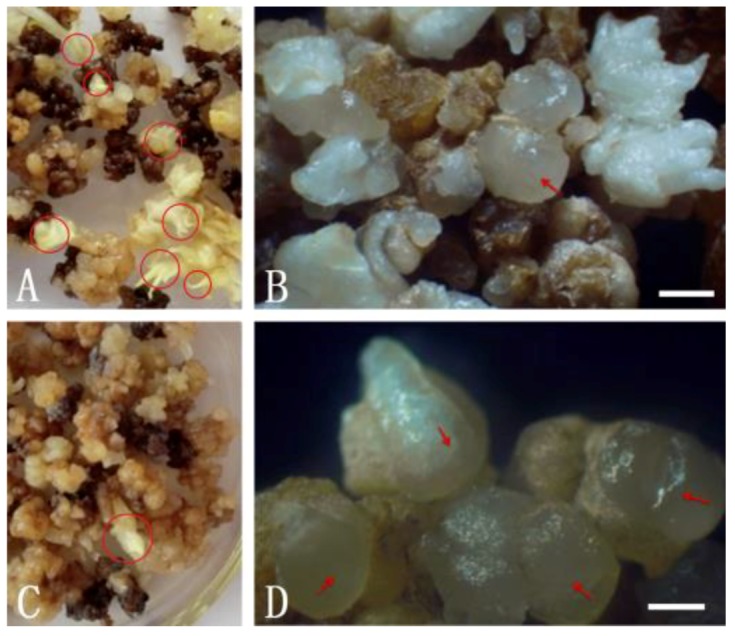
Effect of methylation inhibitor 5-azaC treatment on somatic embryo induction. (**A**,**B**) Display embryos without 5-azaC pretreatment, and (**C**,**D**) display embryos with 5-azaC pretreatment, and all panels are representative images of embryos 30 days after somatic embryo induction. Arrows indicate globular embryos. Circles indicate adventitious shoots. Scale bar = 400 μm.

Somatic embryogenesis of pineapple proceeds through five stages: protoembryo, globular embryo, pear-shaped embryo, bamboo-shaped embryo, and mature embryo. Somatic embryos develop to the stage of globular embryo after somatic embryo induction of 30 days [18]. Somatic embryogenesis is often accompanied by germination of adventitious buds, which affects the efficiency of SE. To analyze the effect of 5-azaC on the process of SE in detail, callus in suspension culture were treated with 5 μmol·L^−1^ 5-azaC liquid MS basic medium for 5 days, or left untreated, and then somatic embryo induction was performed. We then analyzed the somatic embryos 30 days later to determine if/how 5-azaC treatment affects the induction process. The results showed that 5-azaC at 5 μmol·L^−1^ significantly inhibited adventitious bud differentiation and increased the number of globular embryos (Figure 5). Specifically, with 5-azaC pretreatment, the amount of globular embryos was 22.15 per gram, which was twice that of the untreated control, with much lower adventitious bud differentiation (Figure 5B,D). Thus, pretreatment with 5-azaC promotes SE.

## 4. Discussion

The formation of plant embryos (zygotes and somatic embryos) involves the rearrangement of large numbers of cells [20,21,22,23,24]. Low levels of DNA methylation during this complex process are closely related to the morphogenesis of embryos and the acquisition of embryonic capacity [25]. For example, short-term DNA demethylation occurs after pollination of the ovules of tadpoles [26]. DNA demethylation also occurs in early stages of embryogenesis in *Brassica napus* and barley microspores [27,28]. In microspores, low levels of DNA methylation are associated with transcriptional activation and cell totipotency of related genes [27,28,29]. This study found significantly lower methylation levels in the CpG islands of the 5′ upstream regulatory region of *AcSERK1* in embryonic callus compared to non-embryonic callus. This finding is consistent with results between specific development stages of somatic embryos and zygotes [17,25,27], but has not previously been described in CpG islands of a specific gene promoter, like *SERK1*. This is because most studies of *SERK1* expression do not address epigenetic regulation, although this is important for gene expression and the transition of somatic cells to embryo cells [25,26,27,28,29]. Additionally, the absence of CpG islands in *AcSERK2* and *AcSERK3* suggests that their gene regulation may occur via a different epigenetic regulation mechanism than *AcSERK1*, where we did find CpG islands that were differentially methylated in embryonic versus non-embryonic callus. We went on to show that methylation inhibition also affects *AcSERK1* expression levels, and somatic embryo induction. We cannot rule out that methylation of another gene besides *AcSERK1* is responsible for the increase in *AcSERK1* expression, or the acceleration of SE, as 5-azaC inhibits DNA methylation genome-wide. However, we consider that methylation of the *AcSERK1* promoter is likely to be involved at least in part, given its known role in this process [30].

Recently, in a related study on the induction of microspore embryogenesis, 5-azaC was shown to promote the induction of embryogenesis [31]. Consistent with this result, in our study, when the concentration of 5-azaC was low (0~5 μmol·L^−1^), the expression level of the *AcSERK1* gene generally increased with increasing concentration. However, at higher concentrations (>50 μmol·L^−1^), this relationship becomes reversed, and the expression levels decrease. *AcSERK1* is a marker gene for pineapple SE, and its expression level in an individual plant corresponds with its capacity to undergo SE, i.e., the early embryonic induction phase of SE [14]. Thus, this result of increasing and decreasing *AcSERK1* transcription indicates that low levels of demethylation increase a plant’s capacity for SE, and higher levels decrease it. Supporting this interpretation is a study of SE in woody plant leaves, showing that the global DNA is demethylated, and the appearance of demethylation reflects the potential for SE [32]. Thus, the reason why the *AcSERK1* expression pattern does not consistently increase with increasing concentrations of the methylation inhibitor may be related to the effect of methylation inhibitors on the phenotype. This has been shown in carrots, where low concentrations of 5-azaC promote SE, while high concentrations (>4.1 μmol·L^−1^) inhibit SE [29]. In pineapple SE, here we show that 5-azaC at 5 μmol·L^−1^ can significantly inhibit adventitious bud differentiation and increase the number of globular embryos (i.e., promote SE). So, 5-azaC at low concentrations (≤5 μmol·L^−1^) promotes SE, whereas we have shown that at high concentrations (≥50 μmol·L^−1^), it inhibits SE in pineapple [33], consistent with the results in carrot [34]. Thus, we propose that 5 μmol·L^−1^ 5-azaC for 5 days is the optimal pretreatment protocol to promote somatic embryogenesis, and it is the concentration that maximizes the expression of *AcSERK1*. However, the effect of 5-azaC toxicity on expression and SE requires further study.

Methylation regulates promoter activity, which likely influences the expression of *SERK1*. The activity of the *SERK1* promoter has embryogenic cell specificity in pineapple, especially during development to the globular embryo stage [35]. In this study, we found that CpG islands in the *SERK1* promoter are demethylated in embryonic callus. We assume this is why the gene expression level was increased in embryonic callus. The pretreatment with a methylation inhibitor verified the effect of demethylation on SE ability. Pretreatment with methylation inhibitors may promote the transformation of more nonembryonic callus to globular embryos during embryogenic induction. Thus, controlling the methylation level of the pineapple *SERK1* promoter may increase the rate of SE. Our methylation analysis of CpG islands in the pineapple *SERK1* promoter will help to decipher the likely complex epigenetic regulation mechanisms of SE, and thereby improve the efficiency of pineapple SE and in vitro propagation.

## Figures and Tables

**Table 1:** Primers used for quantitative real-time PCR (qPCR).

Primer Name	Primer Sequence	References
*AcSERK1*_F_	5′-AACCGTTCTACTTTACTGGCTTTGG-3′	Ma et al., 2012 [12]
*AcSERK1*_R_	5′-GCATCTCTTCAGCGTAAGGGTAAT-3′	
*β-actin*_F_	5′-CTGGCCTACGTGGCACTTGACTT-3′	
*β-actin*_R_	5′-CACTTCTGGGCAGCGGAACCTTT-3′	

**Table 2:** Specific primers for CpG island amplification of *AcSERK1* promoter.

CpG Island	Forward	Reverse	Product Size
CpG-1	AAAAAGAAGATATTTGGGAACTTTTG	CTAATTTATTTCTTTATTATCTTCTT	392 bp
CpG-2	GGGGGAAAAAAGTAGAAG	CATTGCCGCCGCCGCGAGCTCCGCCG	292 bp
